# The ant’s weapon improves honey bee learning performance

**DOI:** 10.1038/s41598-023-35540-7

**Published:** 2023-05-24

**Authors:** Antonia Bachert, Ricarda Scheiner

**Affiliations:** 1 Behavioral Physiology and Sociobiology, University of Würzburg, Am Hubland, 97074 Würzburg, Germany; 2 Institute of Pharmacology and Toxicology, University of Gießen, Schubertstraße 81, 35392 Gießen, Germany

**Keywords:** Animal behaviour, Animal physiology

## Abstract

Formic acid is the main component of the ant’s major weapon against enemies. Being mainly used as a chemical defense, the acid is also exploited for recruitment and trail marking. The repelling effect of the organic acid is used by some mammals and birds which rub themselves in the acid to eliminate ectoparasites. Beekeepers across the world rely on this effect to control the parasitic mite *Varroa destructor*. Varroa mites are considered the most destructive pest of honey bees worldwide and can lead to the loss of entire colonies. Formic acid is highly effective against Varroa mites but can also kill the honeybee queen and worker brood. Whether formic acid can also affect the behavior of honey bees is unknown. We here study the effect of formic acid on sucrose responsiveness and cognition of honey bees treated at different live stages in field-relevant doses. Both behaviors are essential for survival of the honey bee colony. Rather unexpectedly, formic acid clearly improved the learning performance of the bees in appetitive olfactory conditioning, while not affecting sucrose responsiveness. This exciting side effect of formic acid certainly deserves further detailed investigations.

## Introduction

The parasitic mite *Varroa destructor*^1^ has been shaking pollination and honey industries for decades since it spread from its original host, the Asian honey bee (*Apis cerana*) to the Western honey bee (*Apis mellifera*)^2^. The mite is a serious pest of honey bees and a major cause of colony losses worldwide^3,4^. Varroa mites harm the bees by feeding on their adipose tissue and cellular components^5^. In addition, the mite is a vector of harmful bee viruses like the Deformed Wing Virus (DWV)^6^ and often the Acute Bee Paralysis Virus (ABPV)^7^. Global warming has led to longer spring and fall brood rearing periods and therefore an increased mite population, because the mites only reproduce in brood cells^8^. A wide range of chemicals used to control *Varroa destructor* are available worldwide, though not all products are registered in every country^9^. One varroacide frequently used by beekeepers in Europe and North America is formic acid^9^. It is the second most commonly used varroacide by beekeepers across Europe^10^ and has been used more and more frequently in the US in the last few years^11^. In nature, formic acid is produced by many ant species as their main weapon against small and large enemies^12^. The organic acid is secreted by a gland and not only used for defense but also employed to mark ant trails. Some mammals and birds are known to “bathe” themselves in formic acid to reduce ectoparasites and protect themselves from infestations^13^.

Formic acid is highly effective against Varroa mites by affecting mitochondrial cytochrome C oxidase, an enzyme of the mitochondrial respiratory chain^14,15^. Binding of formic acid to this enzyme assumedly impairs the mitochondrial electron transport chain of the Varroa mite^15–17^, leading to an inhibition of cellular respiration and consequently to acidosis of the body^18^. Formic acid is currently considered the most reliable treatment against Varroa mites, because it offers numerous advantages over synthetic and alternative agents^19–21^. It is applied systemically to the bee colony in liquid form by means of evaporators or impregnated gel strips. But formic acid has some side effects on the bees. It can increase the mortality of bees and brood^20,22,23^. The recent results of Genath et al.^14^ suggest that the higher sensitivity of the younger larval stages to formic acid compared to the newly hatched worker bees is because of a lower detoxification capacity due to a reduced endowment with appropriate detoxification enzymes compared to adult bees. In addition, the use of the acid can lead to the loss of queen or queen acceptance and thus to silent reversion^24^. Pollen and nectar foraging behavior was reduced following treatment with high doses (LD50) of formic acid^25^. Surprisingly, next to nothing is known about side effects of formic acid on honey bee sensory perception or cognition.

Learning is an integral part of honey bee behavior^26^. Bes are central place foragers, and the success of individual foragers in finding sufficient food sources is essential for the survival of the entire colony. The great learning capacity of the honey bee is reflected by its long-standing use as a model organism in neurobiology^27^. Honey bees learn the scent, color and shape of flowers in order to memorize the most rewarding plants^28^. They forage within a range of several kilometers and have excellent place memories^29^. In addition, honey bees have an excellent nestmate recognition, which is important during times of sparse nectar flow and robbery. Their longtime memory for hive mates lets them easily distinguish nestmates from non-nest members^30^. Honey bees are masters in differentiating different sugar solutions by their taste organs, with a particular role of the antenna^31^. Individual perception and responsiveness to sugars is not only a major component of social organization^32^ but strongly correlates with their appetitive learning performance^33^.

We have here investigated for the first time how a formic acid treatment aimed at reducing the Varroa mite load of a honey bee colony affects sucrose responsiveness and cognitive skills of honey bees. The effects of evaporated formic acid were studied on foragers and on bees which were in the embryonal development at the beginning of the treatment, thus representing brood experiencing the treatment during the entire developmental phase. The proboscis extension response (PER)^34^ was employed to study responsiveness to sucrose. Differential olfactory conditioning was used to assess cognition^34,35^.

## Material and methods

**Figure 1:**
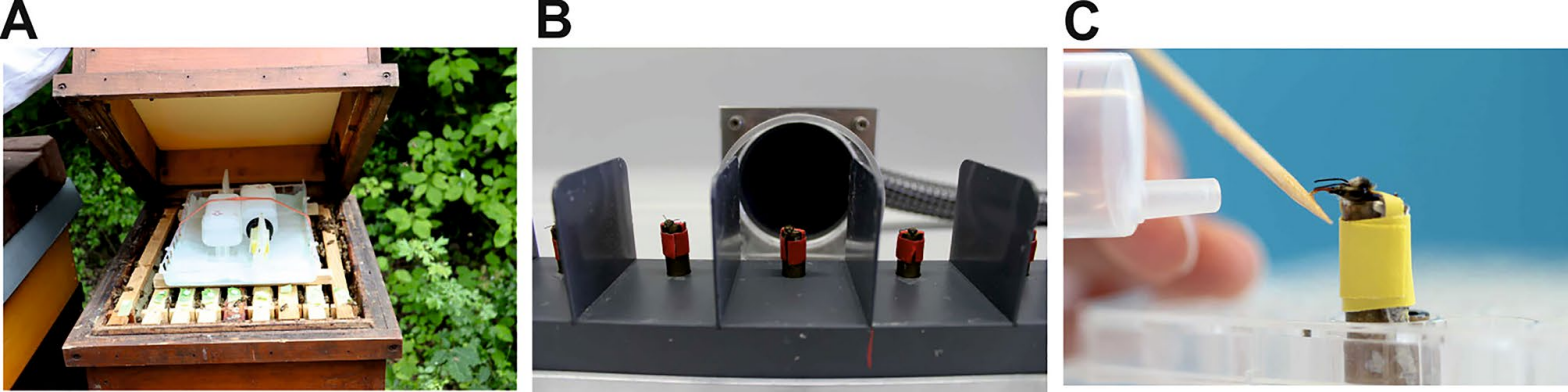
(**A**) Formic acid treatment with the Nassenheider professional®—long term evaporator. The evaporators fixed with a rubber band are placed on the upper frame of the colony and are covered by an empty frame constricted with styrofoam. (**B**) Bee mounted for differential olfactory conditioning of the proboscis extension response (PER). To avoid odor contamination, the animals were placed individually in a fixture in front of a fume hood. (**C**) Olfactory conditioning. The fixed honey bee is stimulated at her antennae with an odor (CS+ or CS−). Either the reward with sugar water (US+) or the punishment with quinine (US−) is presented overlapping in time. Honey bees showing a PER are allowed to drink the respective solution.

Experiments were conducted from June to July 2022 at the University of Würzburg. Two two-roomed, queen-right colonies were placed side by side in Zander magazine hives within the departmental apiary. The queens of the two colonies were sisters to reduce genetic effects on sensory responsiveness and learning performance^36^. The colonies were divided into two experimental groups. One of them was treated with 460 g of 60% formic acid with the Nassenheider Professional long term evaporator (Joachim Weiland, Werkzeugbau GmbH & Co KG, Hoppegarten, Germany) for 14 days (Fig. 1A), the other was an untreated control colony. Since the experiments were performed in mid season, the natural Varroa load of the control colony was average and comparable to that of the treatment group.

This dosage of formic acid was selected, because it was recently shown to result in an effectiveness of 90% as required by the Committee for Medicinal Products for Veterinary Use (CVMP)^22,35^. Before the start of the experiment, combs of both colonies were marked with the brood stage to ensure comparable egg stages across treatment groups. The youngest brood was exposed to formic acid for at least 14 days. Due to the described brood mortality^14,18^ as a side effect of formic acid evaporation, the colony with the largest area of capped brood on the combs was selected for treatment. We applied 60% formic acid (Andermatt Biovet GmbH Lörrach, Germany). The evaporator was placed on the top support with one-cm-thick wooden slats and an empty frame constricted with styrofoam (42 × 85 cm). The evaporation principle with four wicks ensures an almost constant volume flow of formic acid during the entire treatment period. Two horizontally overhanging wick inserts were placed in the bottom tray of the evaporator. In addition, the two wick holders were each fitted with a size three wick. The evaporators were attached by means of a rubber band. Only 290 ml fit into one bottle of the evaporator, according to the manufacturer’s specifications. We therefore used two bottles of formic acid in order to reach the highly effective dose of formic acid, according to CVMP^22^. After detailed consultation with the manufacturer, we decided to install one size three wick in each of the two wick holders and also to install two horizontally overhanging wick inserts in the base tray of the evaporator. The two bottles had to be secured with a rubber band, as there is only a single stand base. Throughout the treatment period, the flight holes were open and bottom slides were inserted.

### Age groups and treatment durations

Treatment with formic acid lasted for 14 days, as prescribed by the manufacturer. To differentiate possible effects of formic acid on bees depending on treatment duration, we grouped (1) outbound foragers and (2) young hive bees.

- (1) Outbound foragers
  In adult foragers we studied the treatment durations: (1) one to three days after onset of treatment, (2) seven to nine days after onset of treatment and (3) 13–14 days after onset of treatment.
  The bees were collected at the flight holes by using glass vials sealed with foam. The foragers were entirely out-flying foragers and no distinction was made between pollen and nectar collectors. Since they were out-flying foragers, it is assumed that their age was between 21 and 30 days.
- (2) Young hive bees
  In young hive bees we used the groups: treatment with organic acid from the egg stage over a period of 14 days. Conditioning was done at the age of (1) 8–15 days, (2) 16–23 days and (3) 24–27 days.
  Regular colony inspections were carried out and the brood was removed from both colonies two days before the calculated hatching date. Hatching took place under controlled conditions in an incubator (temperature: 35 °C, humidity: 50%). On three consecutive days, about 50 bees per treatment hatched within 24 h and were placed in separate cages (7.8 × 5.0 × 8.2 cm), marked with date and treatment. The newly emerged bees were reared in a different incubator (temperature: 30 °C, humidity: 50%). They were fed ad libitum with 30% sugar water solution and a 1 g "pollen ball" prepared from 20 g pollen and 5 g liquid food syrup (Apiinvert®). Sugar water and pollen ball were replaced daily. Training began when the bees were at least eight days old, because younger bees generally learn poorly^37^. The young hive bees were collected from the particular cages by using glass vials and sealed with foam.

### Both groups

To achieve immobilization, the glasses were shortly placed on ice. After anesthesia, they were fixed in holders with a strip of textile tape between head and thorax and a second strip across the abdomen^34,38^. The mouthparts and antennae were free to move. After a period of two hours for acclimatization, the behavioral experiments started. This duration was important to increase the learning motivation of the bees, because fed bees do not learn well due to a low sucrose responsiveness^39^.

### Measuring sucrose responsiveness

Prior to olfactory conditioning, individual sucrose responsiveness was determined of using the PER. Sugar water solutions were prepared in the following concentrations (0.1%; 0.3%; 1%; 3%; 10%; 30%). The solutions were presented in ascending order to both antennae using a toothpick. The waiting time between each sucrose concentration was two minutes to avoid intrinsic sensitization^34,40^. After each stimulation, it was recorded whether the bee showed a PER. For number of bees tested for sucrose responsiveness see Table S1.

### Olfactory conditioning

Only bees showing a PER to 50% sucrose solution were selected for the learning experiments, because this solution was used as reward. The odors were 1-nonanol and eugenol (74,278 1-nonanol, E51792 eugenol; Sigma Aldrich, Steinheim, Germany). Five µl of each odor were applied onto a 1cm^2^ filter paper placed into a 20 ml syringe. The odor-air mixtures were presented to the subjects with this syringe. Prior to training, the spontaneous reaction of the bees to the two scents was tested. For this purpose, the bees were placed in front of a fume hood to avoid contamination with the released scents. Only bees not showing a spontaneous response (proboscis extension response, PER) were selected for training (Fig. 1B). The subjects were to learn to associate the odor 1-nonanol (conditioned stimulus, rewarded, CS+) with a 50% sugar water solution (unconditioned stimulus, reward, US+) as a reward and associate the odor eugenol (conditioned stimulus, punished, CS−) to the punishment with quinine solution (unconditioned stimulus, punishment, US−) (quinine: 60 mM^41^). In each of the five conditioning trials, the odors were presented alternately. Scent exposure totaled eight seconds. By applying slow, steady pressure to the plunger of the syringe, either scent was presented for three seconds. In the following five seconds, depending on the scent (CS+ /CS−), the sugar water or quinine solution was additionally presented to the antennae of the bees using a toothpick and the bees were allowed to drink from that solution for one second, which amounts to approximately one µl (Fig. 1C).

In each training trial, it was recorded whether the bees responded to the CS+ or the CS− with proboscis extension. The sequence of conditioned stimuli was CS+ CS− CS+ CS− … for a total of 10 trials, 5 with each odor.

### Statistics

The response of each bee to a sugar solution or to an odor stimulus during conditioning was recorded as a binary response ("PER" was scored as "1", while "no PER" was scored as "0") and analyzed using logistic regression (generalized estimating equations (GEE) with logit function and binary response variable, SPSS® Statistics (Version 28.0.1.0, IBM®, Armonk, NY USA). Predictor variables were "treatment" and "treatment duration". Logistic regression has been used in several previous studies of sucrose responsiveness and olfactory PER learning^42–44^.

## Results

### Responsiveness to sucrose is unaffected by formic acid

**Figure 2:**
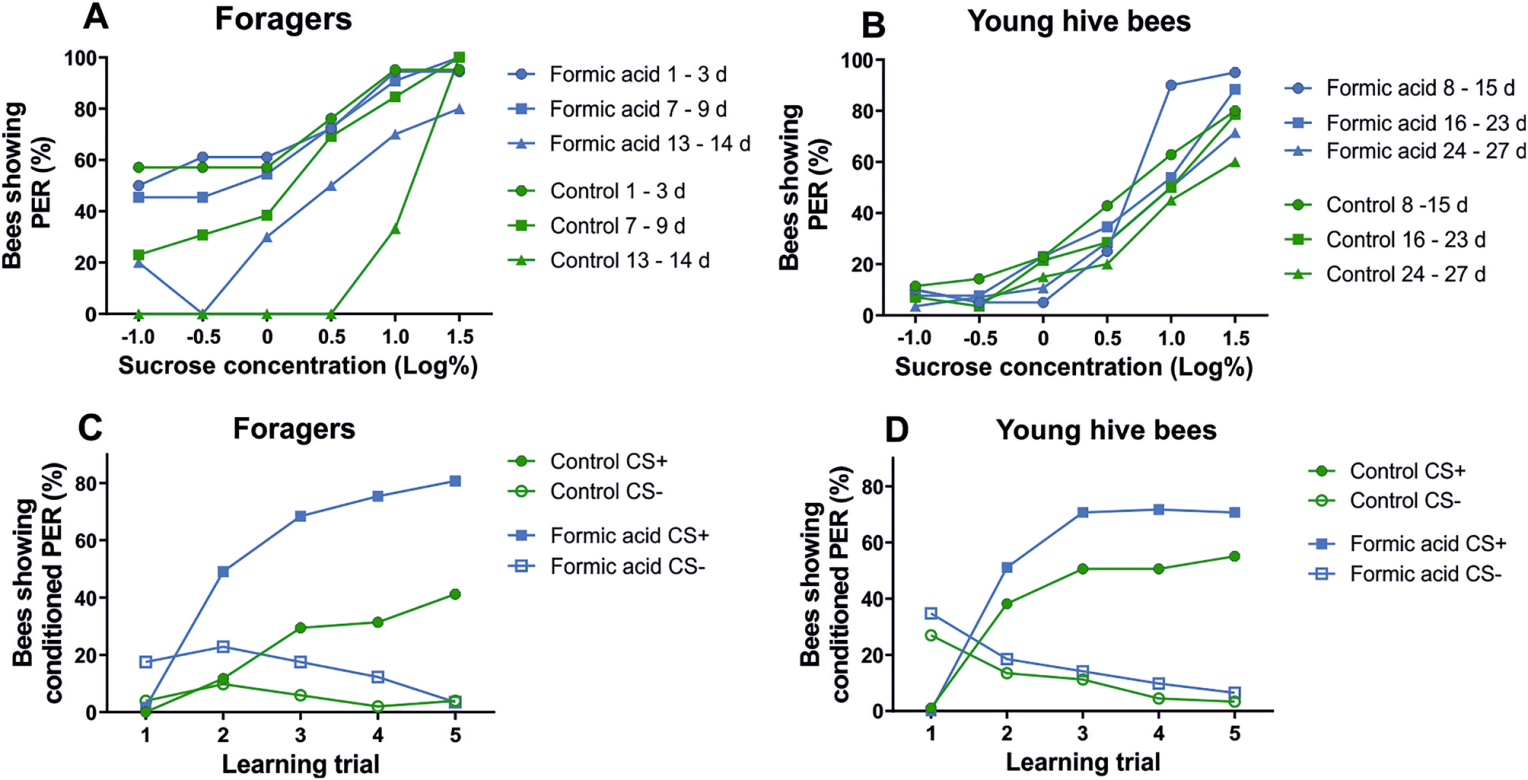
(**A**, **B**) Sucrose-concentration response curves of foragers and young hive bees treated with formic acid for different durations. (**A**) Foragers. (**B**) Young hive bees. Treatment had no effect on sucrose responsiveness in foragers or young hive bees (*P* > 0.05). Treatment duration affected sucrose responsiveness in young hive bees (*P* < 0.05) but not in foragers (*P* > 0.05). For details on number of bees tested and statistics see text. (**C**, **D**) Learning curves to CS+ and CS− of bees experiencing formic acid. The proportion of bees showing conditioned PER increases with the number of learning trials. Both foragers (**C**) and young hive bees (**D**) learned to respond with PER to the CS+ and not to respond to the CS−. Formic acid treated bees showed a significantly higher response to the CS+ than the control group (foragers: *P* < 0.001, hive bees: *P* < 0.01). Learning not to respond to the CS− was affected by formic acid in foragers (*P* < 0.05) but not in hive bees (*P* > 0.05). In both age groups, treatment duration did not affect responses to the CS− (*P* > 0.05).

Treatment with formic acid did not affect sucrose responsiveness in foragers (Fig. 2A; χ^2^_1_ = 0.38, *P* > 0.05, GEE) or young hive bees (Fig. 2B; χ^2^_1_ = 0.28, *P* > 0.05, GEE). Responsiveness generally increased with increasing sucrose concentrations. This was independent of whether bees had experienced formic acid or not. Treatment duration had a small but significant effect on sucrose responsiveness in young hive bees (Fig. 2B, χ^2^_2_ = 8.68, *P* < 0.05, odds ratios: 8–15 days: 0.39, 16–23 days: 0.53, 24–27 days: 1, GEE) but not in foragers (Fig. 2A; χ^2^_2_ = 4.44, *P* > 0.05, GEE).

### Differential olfactory learning performance

Formic acid significantly improved learning of the CS+ of both foragers (Fig. 2C; χ^2^_1_ = 26.96, *P* < 0.001, odds ratio: 4.18, GEE) and young hive bees (Fig. 2D; χ^2^_1_ = 7.02, *P* < 0.01, odds ratio: 1.72, GEE). Since “treatment duration” did not affect learning performance of foragers (χ^2^_2_ = 0.29, *P* > 0.05, GEE) or of hive bees (χ^2^_2_ = 5.52, *P* > 0.05, GEE), we pooled the respective data within each age group for visualization of CS+ and CS− learning curves (Fig. 2C, D).

## Discussion

Our results have demonstrated for the first time that formic acid, which ants use as a highly effective chemical defense, can improve the appetitive olfactory learning performance of honey bees. Foragers exposed to formic acid and young hive bees learned the rewarded scent significantly better than the respective untreated control. While a better learning performance normally coincides with a higher responsiveness to sucrose^33,44–46^, this was not the case in our experiment.

We therefore hypothesize that formic acid ultimately targets signaling cascades involved in the formation of the association between the sugar water reward and the conditioned stimulus, thereby leading to a better learning performance. Associations between CS and US are built in the mushroom bodies of the honey bees, i.e., we expect formic acid to either directly act there or to induce e.g. the release of octopamine there. The neurotransmitter and neurohormone octopamine^47^ was shown to mediate the reward signal in olfactory PER conditioning^48^ and increasing octopamine titers in the brain can improve appetitive learning in honeybees^49^.

The central role of biogenic amines as neuromodulators in honeybees has been demonstrated during habituation, sensitization, and associative olfactory learning^47^. The first evidence for the significant influence of octopamine comes from electrophysiological experiments by Hammer^50^ on the VUMmx1 neuron (ventral unpaired neuron no. 1 of the maxillary neuromere) of the SEG (subesophageal ganglion). Depolarizations of VUMmx1 may substitute for the reward property of the unconditioned stimulus (US) during classical conditioning^50^, probably due to the depolarization-induced release of octopamine. In addition, octopamine injections into antennal lobes or mushroom body calyces can substitute for the US during classical conditioning^48^, and suppression of octopamine receptor gene expression by RNA interference can impair acquisition and retrieval of appetitive odor learning^51^.

Most likely, formic acid will not directly target the mushroom bodies but induce some form of stress, leading to octopamine release in the mushroom bodies. Gunes et al.^52^ demonstrate that formic acid can increase stress levels in honey bee, measured by a changed expression of heat shock genes in the bee brain. Stress reactions of bees directly after formic acid treatment also became evident in our experiment. Two hours after the application of formic acid, bees accumulated at the flight hole to escape the evaporating acid. Honey bees release the neurotransmitter and neurohormone octopamine in response to acute stress^53^. A stress-induced release of octopamine in the mushroom bodies might therefore be responsible for the better learning performance of honey bees observed in our experiments, while not affecting sucrose responsiveness.

Alternatively, formic acid may have directly affected the perception and/or evaluation of the olfactory conditioned stimuli. However, in that case we would have expected a stronger differentiation between CS+ and CS−, which was only the case in hive bees, while foragers treated with formic acid responded more frequently to the CS− than control bees, displaying a larger degree of generalization between CS+ and CS− and a poorer performance during reversal learning. This might correlate with a higher value the foragers attach to the former CS+, therefore continuing to respond to the new CS− during reversal learning.

The exact molecular mechanism underlying the action of formic acid is still largely unknown in both honey bees and Varroa mites. Formic acid can differentially affect expression of genes in the detoxification pathway^54^. In support of this finding, Gashout et al.^55^ showed that formic acid can inhibit the gene CYP9Q3, which is partly responsible for detoxification. *Defensin-1*, a gene involved in the immune response, is upregulated by treatment with formic acid. However, nothing is known about a connection between these genes and octopamine release in the central brain. This aspect certainly deserves a more detailed experimental analysis.

Another reason for the better learning performance of bees after formic acid treatment might be related to metabolism. Saturation has a decisive influence on appetitive learning performance in honey bees^56^. Hungry bees generally perform better than satiated bees. We hypothesize that formic acid may affect metabolism in honey bees either directly or through inducing energetic stress in the bees similar to that observed after artificial infection with *Nosema ceranae*^57^, which results in higher levels of energy demand. Formic acid treatment might also directly affect metabolism by disrupting nutrient absorption, leading to metabolic imbalances and nutritional deficiencies. In support of this assumption, formic acid was shown to induce a downregulation of genes associated with lipid metabolism^58^.Our results show that formic acid can improve learning performance in honey bees. Chemical substances used in the human field to increase performance and learning ability include the purine alkaloid 1,3,7-trimethyl-3,7-dihydro-2H-purine-2,6-dione, i.e. caffeine. It has an effect on chemical messengers mediating communication between brain cells and nerve pathways and inhibits cAMP phosphodiesterase^59^. It further acts as an antagonist at adenosine receptors, leading to dopamine release and activation of further brain areas^60^. This, in turn, improves learning performance. A study by Si et al.^61^ investigated the effects of caffeine on olfactory and visual learning in the honey bee (*Apis mellifera*) and confirmed the effects of the stimulant in insects. It will be an exciting question of whether formic acid can have similar positive effects on associative learning performance in other insects and invertebrates.

In our experiments, we used two colonies of similar size with sister queens. Since a queen naturally mates with multiple drones^62^, worker bees of both colonies represented a mixture of different genotypes, which might have affected their appetitive learning performance additionally ^35,63,64^. Intriguingly, bees of both treatments did not differ in their sucrose responsiveness, which is a major determinant of appetitive learning performance^33,45,65^. Since we cannot exclude an effect of genotype on responsiveness to formic acid treatment, future studies should include genotype and colony as additional factors.

## Conclusion

Honey bees exposed to formic acid learn significantly better than untreated controls, regardless of age or treatment duration. We therefore expect a positive effect on foraging efficiency in colonies treated with formic acid, since learning is an integral part of central place foraging performed by honey bees. This might be a positive side effect of formic acid treatment, possibly through releasing octopamine in the central brain in response to treatment stress. Ultimately, a higher efficiency of honey bee foragers whose colony had been treated with formic acid might compensate for the loss of worker bees resulting from high brood mortality during treatment.

## Supplementary Information

Supplementary Information.

## Data Availability

All datasets generated during the current study are available from the corresponding author on reasonable request.
