# Supplementary material for: The ant’s weapon improves honey bee learning performance
Source: Sci Rep. 2023 May 24;13:8399. doi: 10.1038/s41598-023-35540-7 (PMC10209135; doi:10.1038/s41598-023-35540-7)
Supplement: Supplementary file 1 — Supplementary Information. [file 41598_2023_35540_MOESM1_ESM.docx]

**Online supplement**

**Table S1.** Number of bees tested for sucrose responsiveness for each treatment duration, treatment and age group.

| **Foragers** | | | |
| --- | --- | --- | --- |
|  | Bees tested for sucrose response per treatment duration | | |
|  | 1 – 3 d | 7 – 9 d | 13 – 14 d |
| Formic acid | 18 | 22 | 17 |
| Control | 21 | 19 | 11 |
| **Young hive bees** | | | |
|  | Bees tested for sucrose response per treatment duration | | |
|  | 8 – 15 d | 16 – 23 d | 24 -27 d |
| Formic acid | 26 | 32 | 34 |
| Control | 35 | 28 | 26 |

**Table S2** Number of bees tested for associative learning for each treatment duration, treatment and age group.

| **Foragers** | | | |
| --- | --- | --- | --- |
|  | Bees tested for learning per treatment duration | | |
|  | 1 – 3 d | 7 – 9 d | 13 – 14 d |
| Formic acid | 18 | 22 | 17 |
| Control | 21 | 19 | 11 |
| **Young hive bees** | | | |
|  | Bees tested for learning per treatment duration | | |
|  | 8 – 15 d | 16 – 23 d | 24 -27 d |
| Formic acid | 31 | 36 | 26 |
| Control | 37 | 31 | 21 |
